# Effects of aspect on phenology of *Larix gmelinii* forest in Northeast China

**DOI:** 10.1038/s41598-022-26712-y

**Published:** 2022-12-22

**Authors:** Chunyuan Dong, Rongrong Qiao, Xueli Chang

**Affiliations:** 1 School of Resources and Environmental Engineering, Ludong University, Yantai, 264025 China; 2 School of Life Sciences, Nanjing University, Nanjing, 210000 China

**Keywords:** Climate sciences, Ecology, Environmental sciences

## Abstract

The response of vegetation phenology to global climate change is one of the main forms in terrestrial ecosystem change, the study of vegetation phenology is an important complement to the understanding of how global climate change affects ecosystems in multiple dimensions. We selected the distribution area of *Larix gmelinii* in The Greater Khingan Mountains as a case area by eliminating the heterogeneity of vegetation types, with the support of Google Earth Engine platform, we studied the effects of different aspects and land surface temperature (LST) on remote sensing phenology (RSP) that is defined as start of growing season (SOS), end of growing season (EOS) and length of growing season (LOS) respectively in the study area through Normalized Difference Vegetation Index (NDVI) changes. The results showed that SOS advanced in different aspects during the study period, and the advance amplitude of SOS on the east and west aspect was greater than that on the south and north. Except for the east aspect, EOS showed a slight postponed, and LOS was prolonged on all aspects. The latitude difference between 51° and 53° N had no significant effect on *L. gmelinii* in different aspects. LST had an obviously direct effect on the RSP of *L. gmelinii* in different aspects, and the effect of LST on SOS and LOS was significantly greater than that on EOS. The effect of LST on SOS and LOS was significant in April and spring. The main contributor to the increase of LOS was the advance of SOS, while the postponed of EOS has a relatively small contribution to LOS. Due to the redistribution of meteorological factor by aspect, the spatial and temporal heterogeneity of RSP tends to be complex, so determining the same aspect is one of the main ways to reduce the phenological heterogeneity in the study of vegetation RSP.

## Introduction

Vegetation phenology is a key attribute of ecosystem function, especially in the context of global climate change having a clear impact on the human living environment^1^. As sensitive indicators of global warming, Changes in the timing of land surface temperature (LST) is about 0.2 °C/10a at the global level in the past decades^2^, while that in China is 0.14 °C/10a; However, it reaches 0.26 °C/10a in arid northwest China^3^. The warming trend has become more evident in recent years, such as January 2020, which broke the global average temperature record and became the warmest January since meteorological records began in 1880^1^. Extreme climate change brings numerous uncertainties to natural ecosystems, it may be manifested in the changes of the interactions between different species and nutrient levels, community composition, and ecosystem equilibrium status in the community^4,5^. Plants as ecosystem producers are the most sensitive to climate change, of which phenology is one of the most important indicators, plant phenology is the phenomenon of periodic changes in plants under the influence of natural conditions such as climate, soil and topography. Plant phenology research mainly includes two approaches, one is through observing and recording the periodic growth and development rhythm of plants such as germination, leaf spreading, flowering, fruiting, leaf falling, withering and wilting at the species scale^6^, the second is remote sensing phenology (RSP), which use various remotely sensed vegetation indexes to determine the start of growing season (SOS), end of growing season (EOS) and length of growing season (LOS) et al. at the regional scale^7^.

In terms of the development of plant phenology research methods, the German botanist Hoffmann established a phenological observation network from the 1890s onwards, conducting a discipline of observation for 40 consecutive years on 34 dominant plants in Central Europe; In 1918, the American forest entomologist Hopkins proposed the biological climate law of the spatial distribution of phenological phenomena in the temperate regions of North America; As the founder of modern phenological phenology in China, Kezhen Zhu established a phenological observation network in 1934 as the beginning of modern phenological observation in China^8,9^. Since the 1950s, due to the expansion of the phenological observation network of many countries, phenological data have become more abundant, such as the China Phenological Observation Network (CPON), the European Phenology Network (EPN), the United Kingdom Phenology Network (UKPN), the National Phenology Network of the USA (USA-NPN) and so on. Especially, the research methods of phenology have been greatly improved in recent decades, from the traditional field fixed-point observation method to the flux observation method, the digital camera observation method, the simulation method of constructing mathematical models based on a series of observational data, and the RSP determination method developed^10,11^, in which the RSP shows great advantages in assessing vegetation phenology at large scales^12,13^. The main indexes involved in the RSP are Normalized Difference Vegetation Index (NDVI), leaf area index (LAI), and fraction of absorbed photosynthetically active radiation (FAPAR) and fraction of vegetation cover (FVC), etc.^14^.

Typical case researches illustrated that the phenological response of forest vegetation to climate in the high latitude area of the China-Mongolia-Russia ecological corridor found that SOS showed an advanced trend, EOS showed a delayed trend, and the factors affecting EOS were temperature and snow line^15^. Lara Carlos et al. used the Enhanced Vegetation Index (EVI) to find that RSP in southwestern South America is affected by climatic factors, indicating that it has a tendency to change with latitude^16^. Eduarda M. O. Silveira et al. selected large study area that distribute from tropical rainforests to cold temperate forests in Argentina and fitted the time series harmonics of the EVI for 2018–2019 to analyze forest RSP, and indicated the heterogeneity of RSP in spatial scale^17^. The common point of these studies is that they take into account the influence of vegetation heterogeneity, and different forest vegetation types are selected for study, so as to exclude the effects caused by differences in different vegetation types (grasslands, wetlands, etc.), but almost no consideration is taken into account the re-distribution of climatic factors by terrain. The influence of topography on the factors is a universal phenomenon, as evidenced by the widespread use of the ANUSPLIN software to calculate precipitation contours in many disciplines^18^. However, the influence of the aspect on the vegetation is the most obvious, such as the differences between the snowline^19^ and the tree line^20^ in different aspects are direct evidences. In addition, lots of findings indicated that relationship between plant phenology and LST is the closest^21,22^, the annual growth curve of NDVI is highly positively correlated with the LST change process over the same period^23,24^. Thus, whether the phenological judgment based on NDVI have similar phenomenon in different aspects needs to be studied in detail.

Therefore, based on the identification of the distribution area of *Larix gmelinii*, the study used a variety of MODIS data (or products) from 2000 to 2020 and combined with the collected local meteorological station data to study vegetation phenology. This study aimed to (1) discover the phenological characteristics of different aspects in the *L. gmelinii* forest. (2) Reveal the response regime of different aspect phenological indicators to LST.

## Materials and methods

### Study area

**Figure 1:**
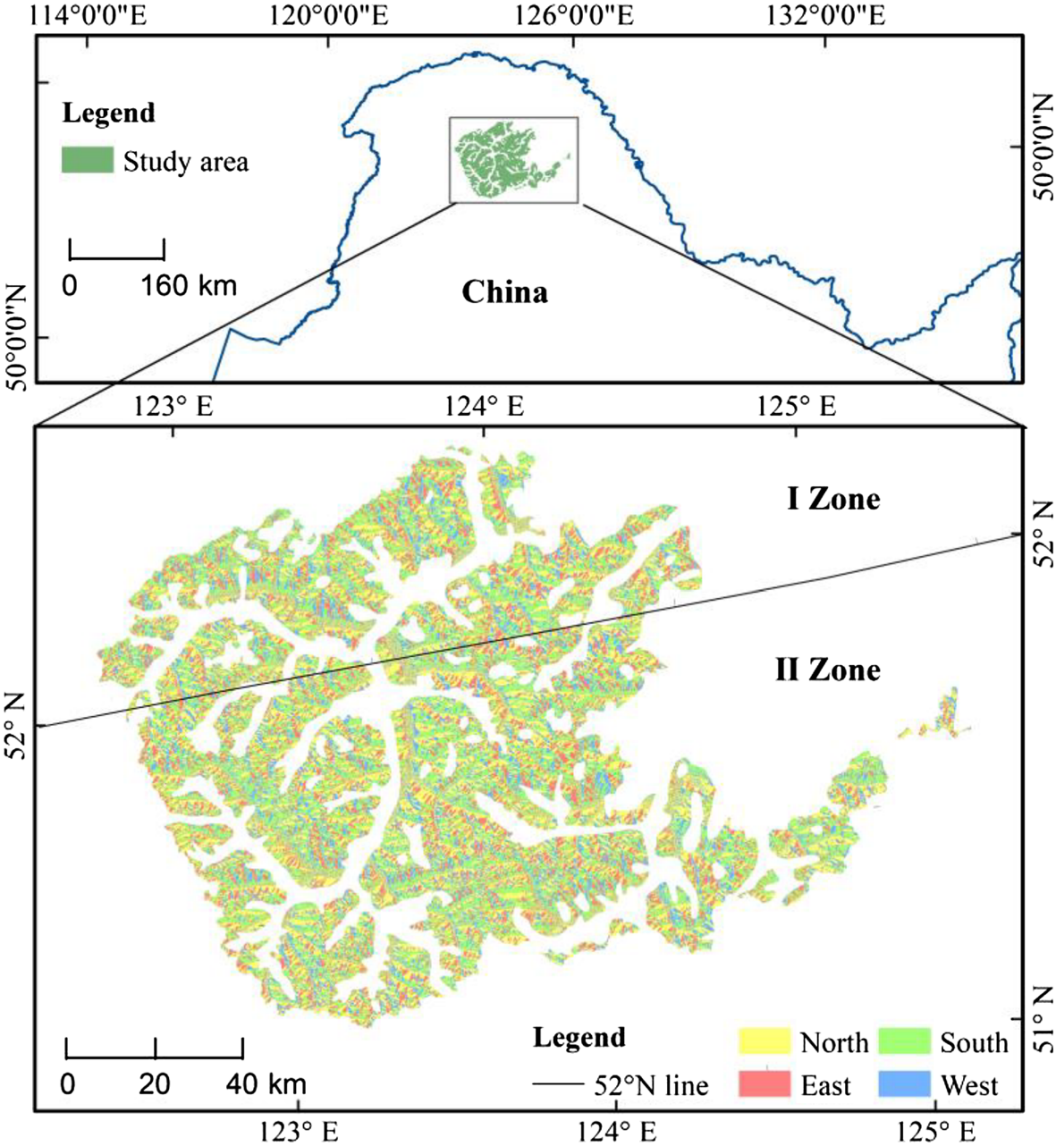
Location and the aspect division of study area.

The study area is located in the Greater Khingan Mountains region of northeast China in central Northeast Asia, with geographical coordinates between 51° 10′ and 52° 20′ N, 122° 30′ E–125° 30′ E (Fig. 1). *L. gmelinii* was the dominant species in the study area, and other major tree species were *Pinus sylvestris* var. *mongolica* Litv., *Betula platyphylla* Suk. and *Picea koraiensis* Nakai, etc. The study area belongs to the cold temperate continental monsoon climate, with long winters and short summers, and there are permafrost areas in the study area. Soil types are dominated by brown coniferous soils and dark brown loam soils^25,26^.

### Data

This study mainly selected the following data: (1) MOD09GQ; (2) MOD13Q1; (3) MOD11A1 V6; (4) Climate change initiative-land cover (CCI-LC); (5) Shuttle Radar Topology Mission (SRTM) (Table 1).

**Table 1:** Data sources used in the study.

Data	Indexes	Temporal resolution	Spatial resolution (m)	Source
MOD09GQ	Red and near-infrared reflectance	1 day	250	modis.gsfc.nasa.gov
MOD13Q1	NDVI	16 days	250	modis.gsfc.nasa.gov
MOD11A1	Land surface temperature	1 day	1000	modis.gsfc.nasa.gov
CCI-LC	Land-cover types	–	300	http://maps.elie.ucl.ac.be/CCI
SRTM	DEM	–	30	http://www.gscloud.cn/

MOD09GQ is used to calculate daily NDVI data, which is corrected for atmospheric conditions such as gas, aerosol and Rayleigh scattering. MOD13Q1 is used to calculate 16 days NDVI, it calculates the vegetation index based on each pixel, mainly including two vegetation layers. The first layer is the NDVI, and the second layer is the EVI. The temperature data is based on the daily surface temperature and emissivity values provided by MOD11A1 V6 to calculate the monthly average temperature for 21 years. The time resolution of all data is 2000–2020. DEM data uses SRTM data (resolution 30 m) for aspect extraction. In determining the distribution range of *L. gmelinii*, CCI-LC products and vegetation type maps of Inner Mongolia, as well as RSP and LST data of different aspects, were resampled to 30 m to match the DEM data.

### Methodology

#### Determination of the scope and the aspect division

First, according to the Product Climate change initiative-land cover(CCI-LC) attribute definition, the land class with attribute values of 60 and 80 (60 is defined as the distribution area of deciduous forest and broad-leaved forest with a coverage greater than 15%; 80 is defined as coniferous and deciduous forest distribution areas with a coverage greater than 15%) were selected to obtain a total forest cover area of 131,101.9 km^2^. Secondly, largest continuous distribution area (> 4 km^2^) of *L. gmelinii* was selected according to the vegetation type map of Inner Mongolia within the forest cover area, with a total area of 13,578 km^2^ (Fig. 1). Finally, the aspect direction is divided into four aspect directions, 45°–135° is east, and 135°–225° is south 225°–315° is west and 315°–45° is north, and the distribution of different aspect areas is shown in Table 2.

**Table 2:** Statistics for different aspect areas.

Aspect	East	South	West	North	Total
Area (km^2^)	2658.0	4412.9	2274.8	4232.3	13,578
Percentage	19.6	32.5	16.7	31.2	100.0

### Mann–kendall (M–K) test

The nonparametric Mann–Kendall (M–K) method is usually used as the base method of detecting trends and abrupt changes and it is widely used in hydrology and meteorology^27,28^. In this study, we employ the sequential M–K test to explore abrupt changes of the *L. gmelinii* phenology, which has been used extensively for detecting the starting point of specific trends. For time series X (with n variables), construct a sequence *S*_*k*_ :

1 $$S_{k} = \mathop \sum \limits_{i = 1}^{k} r_{i} \quad r_{i} = \left\{ {\begin{array}{*{20}l} 1 \hfill & {x_{i} > x_{j} } \hfill \\ 0 \hfill & {else} \hfill \\ \end{array} } \right.\quad j = 1,2, \ldots i.$$

where the values of *r*_*i*_ are denoted by the number of cases *X*_*i*_ > *X*_*j*_ ⁠, *X*_*i *_and *X*_*j*_ are the *i* th (*i* = 1, 2, …, n) and *j* th (*j* = 1, 2, …, i) data values in time series. Then we can create another sequence *UF*_*k*_ ⁠, which is the sequence calculated in the order of time series *X* (*X*_*1*_, *X*_*2*_, … , *X*_*n*_), and it is calculated by Eqs. (2)-(4):

2 $$UF_{k} = \frac{{S_{k} - E\left( {S_{k} } \right)}}{{\sqrt {Var\left( {S_{k} } \right)} }} k = 1,2, \ldots n$$

3 $$E(S_{k} ) = \frac{n(n - 1)}{4}$$

4 $$Var\left( {S_{k} } \right) = \frac{{n\left( {n - 1} \right)\left( {2n + 5} \right)}}{72}$$

where E(*S*_*k*_) and Var(*S*_*k*_) are the mean and variance of *S*_*k*_ respectively. Similarly, we can obtain a retrograde sequence *UB*_*k*_ by calculating from the last data of the time series *X* (*X*_*n*_, *X*_*n*–1_, … , *X*_1_).

A positive *UF*_*k*_ denotes an upward trend while the negative denotes a downward trend. Define a variable *UF*_α/2_ which is the critical value of the standard normal distribution with a probability exceeding α/2, where α is the statistical significance level concerned and is set at 0.05 significant levels (corresponding level is 1.96) in this study. If the absolute value of *UF*_*k*_ is greater than *UF*_*α/2*_, there is a significant trend. The intersection points of the two lines *UF*_*k*_ and *UB*_*k*_ within the confidence interval are the points of abrupt change^29^.

### Phenological determination rules

**Figure 2:**
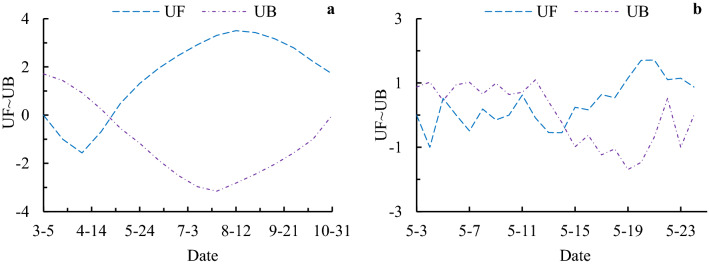
Case Analysis of mutation points in special years (West SOS, 2000) based on MKtest (**a** 16 days; **b** daily).

The double determination rule was adopted for the determination of vegetation phenological indicators. First, the initial determination was made with 16 days NDVI data, and the inflection point (or possible range) of the initial change of NDVI on SOS and EOS in a certain year was roughly determined, and then the secondary mutation analysis was carried out with the data of the first 8 days and 8 days after the primary mutation point, and if there was no mutation point, 1 day was added before and after until the mutation point appears, and the mutation point location (i.e. SOS and EOS) is finally determined. There are two phenomena in the process of determining SOS and EOS based on M–K, one is that *UF*_*k*_ and *UB*_*k*_ intersects only once, and the other is this have more than two times intersections (SOS or EOS). If there is a multiple intersection in the second judgment, the first occurrence of the intersection point is the mutation point (Fig. 2).

In addition, in order to further determine the temporal and spatial change trend of vegetation phenology, this paper uses the mean standard deviation method to delineate the normal fluctuation range, that is, the values above the sum of the mean and the standard deviation are identified as delaying or prolonging the obvious time or area, and the values below the difference between the mean and the standard deviation are identified as ahead or shortened.

### Latitude difference analysis

Since the study area was between 51 and 53 degrees north latitude, it was divided into two sections for the analysis of latitude difference, namely zone I and II from 51° N to 52° N and 52° N to 53° N, respectively. Analysis of Variance (ANOVA) was analyzed, and the significance test threshold was significant at *P* ≤ 0.001, *P* ≤ 0.01, and *P* ≤ 0.05 respectively. The phenological time expression sets January 1 of each year as 1, and so on.

### Sensitivity of RSP to LST

Considering the overlapping relationship between RSP and LST in time spans, the average LST of April, May, Spring (March–May) and year were employed to analyze relationship between SOS and LST, the average LST of September, October, Autumn (September–November) and year were used to EOS and LST, and the average LST of all time spans above were used to LOS and LST, respectively. Aims are to reveal details of the impact of LST on RSP of *L. gmelinii* forest.

All NDVI and LST calculations were done in Google Earth Engine, and other statistical analyses were done in Excel, DPS and SPSS18. The calculation codes of NDVI and temperature are in the supplementary information.

## Results

### Aspect and phenology

Table 3 summarizes the statistical characteristics of different aspect phenological indicators from 2000 to 2020 (Table 3). From the overall comparison with different aspects, SOS was advanced by 1.7 d and 0.8 d on the east and south aspects respectively, and delayed by 0.8 d and 1.8 d on the north and west aspects, respectively. In terms of the coefficient of variation (CV) of SOS, the CV of all aspects except the south aspect is greater than that of the whole. EOS was delayed by 1.6 d and 0.6 d on the north and south aspects respectively, and advanced 1.5 d and 2.3 d on the east and west aspects respectively. The CV of all aspects were greater than the total. LOS showed a prolonged trend except that it was shortened by 4.1 d in the western aspect. The maximum prolonged time was 1.4 d on the southern aspect, while other aspects were smaller. The CV of all aspects was greater than the total CV.

**Table 3:** Statistical characteristics of phenological indicators on different aspects.

Phenology	Statistical description	Aspect				Total
		North	East	South	West	
SOS (DOY)	Mean	115.4^a^	112.9^a^	113.8^a^	116.4^a^	114.6^a^
	Standard deviation	13.6	13.6	13.0	15.3	13.3
	Variable coefficient	0.117	0.123	0.115	0.131	0.116
EOS (DOY)	Mean	279.7^a^	276.6^a^	278.7^a^	275.8^a^	278.1^a^
	Standard deviation	6.5	6.4	6.4	6.5	4.9
	Variable coefficient	0.023	0.023	0.023	0.023	0.018
LOS (Days)	Mean	164.2^a^	163.7^a^	164.9^a^	159.4^a^	163.5^a^
	Standard deviation	14.6	15.4	15.3	17.2	14.4
	Variable coefficient	0.089	0.094	0.093	0.108	0.088

The same letters indicate that there is no significant difference between the different aspects.

From the comparison of different aspects (Table 3). SOS began at the earliest 112.9 (± 13.6) d on the east aspect, that is, late April; and at the latest 116.4 (± 15.3) d on the west aspect, that is, the end of April was close to May; in addition to the east aspect, the first is the south aspect, and the north aspect is slightly earlier than the west aspect. EOS occurred early on the west aspect, averaging 275.8 (± 6.5) d, that is, the vegetation stopped growing at the end of September, and at the north aspect, averaging 279.7(± 6.5) d, and the vegetation stopped growing in early October; secondly, the EOS ended late in the south aspect, and the average value was only 1 day later than that in the north aspect. The longest duration of LOS on the south aspect is 164.9 (± 15.3) d, and the shortest duration on the west aspect is 159.4 (± 17.2) d. Except for the west aspect, the average value of LOS on the other three aspects can reach more than 163 days. The north aspect is slightly shorter than the south aspect, but the average is 0.5 days longer than the east aspect. In addition, the standard deviation of three phenological indicators in the west aspect of the four aspects is greater than that in the other aspects, indicating that the west aspect has the largest dispersion with the mean value.

In terms of different aspects comparison (Table 3), SOS were the earliest for 112.9 (± 13.6) d at east aspect and the latest for 116.4 (± 15.3)d at western aspect; EOS ended earliest for 275.8 (± 6.5) at western aspect and latest for 279.7(± 6.5)d at north aspect. Duration of LOS was maximum to 164.9 (± 15.3) d at south aspect and minimum to 159.4 (± 17.2) d at west aspect.

**Figure 3:**
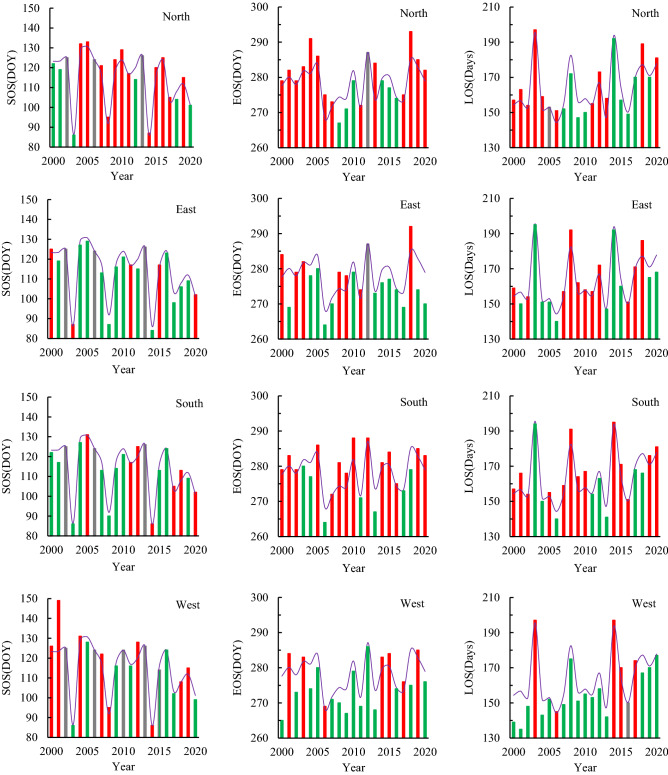
RSP indicators of different aspects (Green represents advance or shortening, red represents delay or extension, and gray represents impervious).

Details of changes in phenological indicators in different aspects were plotted to assess the details from 2000 to 2020 (Fig. 3). On the north aspect, the SOS delayed phenomenon occurred more frequently than advance and account for 12 years and 6 years, respectively. EOS postponed phenomenon also occurred more frequently than advance and account for 14 years and 6 years, respectively. The frequency of prolonged LOS was 11 years more than that of shortened LOS (9 years). On the east aspect, the frequency of SOS advance occurrence was more than the delayed frequency, which is 13 years and 5 years, respectively. The EOS advance phenomenon also occurred more frequently than the delay, which is 13 years and 7 years, respectively. The frequency of prolonged LOS was 11 years more than that of shortened LOS (10 years). On the south aspect, the frequency of SOS advance occurrence was more than the delayed frequency, which is 11 years and 7 years, respectively. The EOS postponed phenomenon also occurred more frequently than the advance, which is 14 years and 7 years, respectively. The frequency of prolonged LOS was 13 years more than that of shortened LOS (8 years). On the west aspect, the frequency of SOS advance occurrence was less than the delayed occurrence, which is 8 years and 9 years, respectively. The EOS advance phenomenon also occurred more frequently than the delay, which is 14 years and 7 years, respectively. The frequency of prolonged LOS was 5 years less than that of shortened LOS (15 years).

### Latitude differences

Table 4 and Fig. 3 show that the latitude difference partitions were no statistically significant differences in the RSP of *L. gmelinii* between 51° and 53°N on different aspects, but there were obvious characteristics from high latitude zone I to low latitude zone II. In the north aspect, SOS and EOS in zone II were both larger than in zone I, more than 0.8 and 0.6 d, respectively; rather than LOS in zone II was more than 0.8 d in zone I. In the east aspect, SOS and EOS in zone II were both smaller than in zone I, less than 0.2 and 0.4 d, respectively; and LOS was also correspondingly less than 0.2 d. In the south aspect, SOS in zone II was greater than that of zone I. 0.7 d, and EOS and LOS were opposite with SOS in zone II less than that of zone I 0.3 d and 0.9 d, respectively. There was no change in SOS in the west aspect of zone I and zone II, while EOS and LOS were 1.7 days higher in the zone II than in the zone I.

**Table 4:** latitude differences of RSP on different aspects.

Phenology	North		East		South		West	
	I	II	I	II	I	II	I	II
SOS	114.0^a^	114.8^a^	113.0^a^	112.8^a^	113.3^a^	114.0^a^	114.4^a^	114.4^a^
EOS	278.0^a^	278.6^a^	275.9^a^	275.5^a^	278.7^a^	278.4^a^	278.5^a^	280.2^a^
LOS	164.0^a^	163.8^a^	162.9^a^	162.7^a^	165.3^a^	164.4^a^	164.1^a^	165.8^a^

The same letters indicate that there is no significant difference between the different zones.

**Figure 4:**
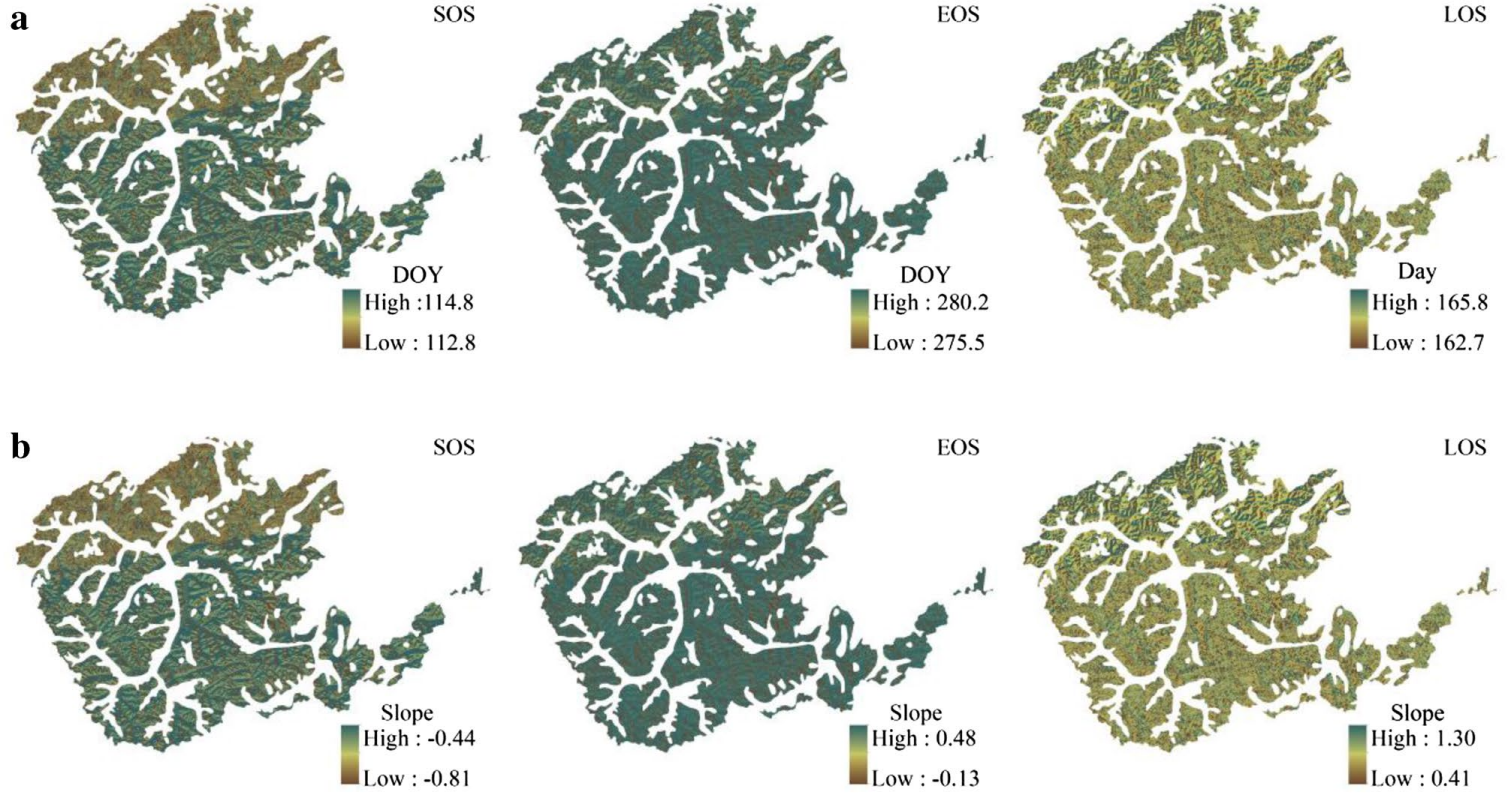
Variation trend of RSP in the Greater Khingan Mountains from 2000 to 2020 (**a** mean value; **b** slope).

**Figure 5:**
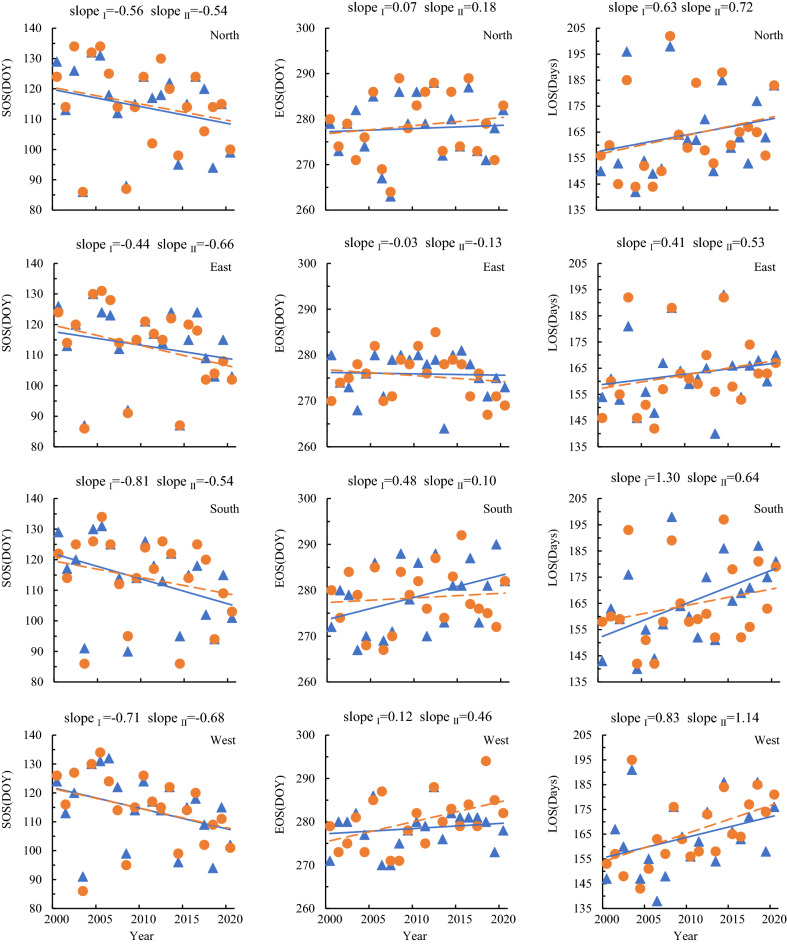
Phenological characteristics of vegetation at the same latitude (Blue triangle, zoneI; Orange circle, zoneII).

In terms of the perspective of the change trend of RSP indicators in different zones during the study period (Figs. 4, 5), the zone I and zone II had no influence on the change trend of each RSP indicator at all aspects, and the positive and negative aspects were consistent, but there were differences in slope size. Among them, there were very smallest differences for all indicators of slope in the north and east aspects, and relatively obvious differences for a few phenological indicators on the south and west aspects. In terms of details, the slope absolute values of the three remote sensing phenological indexes in the south aspect were all larger in the zone I than in the zone II, in which the absolute values of SOS slope were 0.27, EOS 0.38 and LOS 0.66 larger. The slope of the all RSP indicator change on the west aspect was opposite to the southern slope, which is less in the zone I than in the zone II, and the EOS slope of the zone II was more than the zone I 0.34. This indicates that there are zonal differences in the RSP indicators of *L. gmelinii*, and the differences were relatively obvious in the south and west slopes, although this difference did not pass the statistical significance check.

### Effects of LST on vegetation phenology

**Figure 6:**
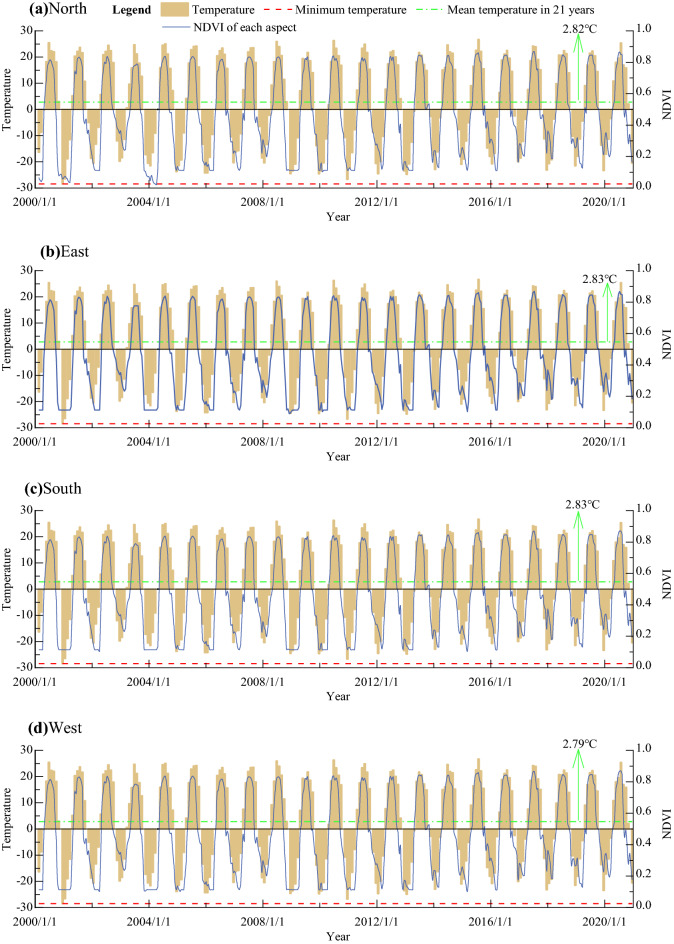
Relationship between NDVI and LST.

Since RSP is determined by NDVI changes, the relationship between LST and NDVI is the basis for analyzing its effect on phenology. Figure 6 shows the relationship between the LST and the NDVI at different aspects, its change rhythms were basically the similar, the higher the LST, the greater the NDVI, and there was a positive correlation between them. During the study period, the lowest mean LST change in the four aspects fluctuated between − 28.46 and − 28.53 °C, and the average LST change was between 2.79 and 2.83 °C.

In terms of LST characteristics at different aspect, average annual lowest LST of the eastern and the south aspect during the study period was about − 24 °C, while the average lowest LST of the west and the north aspect was about − 26 °C, and the average annual LST (2.83 ± 0.92 °C) of the east and the south aspect was also higher than that of the west aspect (2.79 ± 0.93 °C) and the north aspect (2.82 ± 0.93 °C), which indicates that the LST was generally higher in sunlit aspect than in ubac aspect, but the maximum range was only 0.04 °C. This clearly indicates that LST changed little on the total during the study period, but the annual differences were great. The mean coefficients of variation of LST in different aspects were 32.7% on the eastern, 32.7% on the southern, 33.2% on the western and 33.3% on the Northern. Meanwhile, during the study period, the variation range of NDVI were 0.019–0.867 in the northern, 0.020–0.868 in the eastern, 0.022–0.868 in the southern, and 0.021–0.866 in the western in four aspects.

In terms of relationship of LST and the RSP of *L. gmelinii*, Table 5 shows that Spring and April mean LST had a significant negative correlation (*P* < 0.01) for SOS at every aspect; for the annual LST, except the south aspect had negative correlation at *P* < 0.01 level, there was negative correlation at *P* < 0.05 level on other aspects. This indicated that the impact of the related LST indicators on SOS did not differ much on the aspects, whether in the corresponding monthly, seasonal or yearly scales. The EOS related LST had no significant effect on EOS in all aspects, indicating that LST was not the main factor directly affecting the change of EOS. LOS involved all LST selection indicators, but was positively correlated with LST only in spring and April in different aspects (*P* < 0.01), there was no significant correlation with LST indicator in rest time periods. It indicates that LST had a significant effect on LOS in spring and April, while LST in other time periods could promote the change of LOS, but has no significant effect.

**Table 5:** Correlation coefficient between RSP and LST in different aspects and time spans.

Phenological factor	Aspect	Temperature index							
		Average LST in Apr	Average LST in May	Average LST in spring	Average LST in Sept	Average LST in Oct	Average LST in autumn	Average LST from Apr. to Oct	Annual average LST
SOS	East	− 0.784**	–	− 0.647**	–	–	–	− 0.229	− 0.453*
	West	− 0.699**	–	− 0.762**	–	–	–	− 0.215	− 0.477*
	South	− 0.748**	–	− 0.759**	–	–	–	− 0.194	− 0.486**
	North	− 0.763**	–	− 0.801**	–	–	–	− 0.193	− 0.473*
EOS	East	–	–	–	0.090	0.366	0.086	0.299	0.000
	West	–	–	–	− 0.204	0.030	− 0.087	0.230	0.127
	South	–	–	–	− 0.040	0.093	− 0.247	0.332	0.028
	North	–	–	–	0.075	0.408	− 0.127	0.026	− 0.032
LOS	East	0.744**	0.317	0.757**	− 0.354	0.117	− 0.297	0.326	0.399
	West	0.799**	− 0.481*	0.804**	− 0.260	− 0.024	− 0.313	0.278	0.473*
	South	0.785**	− 0.416	0.742**	− 0.415	− 0.009	− 0.381	0.304	0.426
	North	0.654**	− 0.303	0.680**	− 0.363	0.130	− 0.127	0.190	0.424

**At level 0.01 (two-tailed), the correlation is significant.

*At level 0.05 (double-tailed), the correlation is significant.

**Figure 7:**
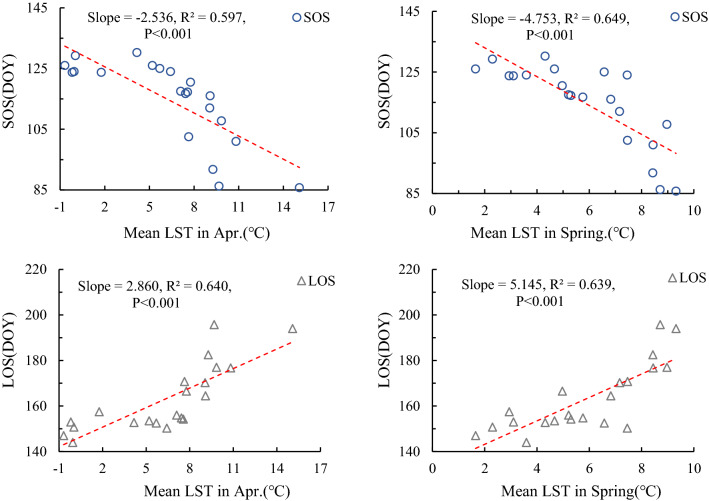
Regression analysis of temperature and phenological indexes.

Since the average LST in April and spring was significantly correlated with SOS and LOS at different aspect, a regression analysis of the relationship between them was performed (Fig. 7). SOS had a significant univariate linear regression relationship with the average LST in April and spring (*P* < 0.001), and both slopes were negative, meaning LST The higher, the sooner the critical LST at the staring growth of vegetation was reached improvingly. Judging from the size of the slope, the average spring LST (March–May) had a greater impact on SOS than April because of the regression slope of average spring LST was 1.87 times that of April. Similarly, LOS had a significant univariate linear regression relationship with the average LST in April and spring (*P* < 0.001), differently both of them was positive, which means that the higher the LST, the later it reaches the critical temperature at the end of vegetation growth, resulting in a prolonged LOS. According to the slope size, it also showed that the impact of average spring LST on LOS was greater than that in April, because the regression slope of average spring LST was April 1.80 times.

## Discussion

Vegetation RSP based on NDVI change is one of the main approach to judge regional environmental changes at a large scale, and its high generalization and relative sensitivity to temperature factors make it have a unique interpretation in screening the impact of global climate change on a certain ecosystem^21–24^. Thus, the distribution area of *L. gmelinii* in The Greater Khingan Mountains in Northeast Asia has become an ideal forest phenology research area after nearly 40 years of forest conservation.

### RSP characteristics at different aspect

At the overall level, the SOS of *L. gmelinii* began on average at 114.6 (± 13.30) d, it showed an advance trend on all aspects in study period. Among them, the sunny aspect (east and south) was on average 0.71 d·a^−1^–1.02 d·a^−1^, and the shady slope (west and north aspect) was on average advanced 0.50 d·a^−1^–0.64 d·a^−1^. SOS advance was closely related to LST, especially in LST anomalous years such as 2003, 2008, 2014 and 2020, the average LST in April was between 8.4 and 10.8 °C, much higher than the average LST (6.3 °C) for the study period, resulting in the SOS being 13.4d–28.7d earlier than the total average one, this phenomenon has been demonstrated in some findings^30^. The change of EOS was relatively small, except for the east 0.07 d·a^−1^ advance, the others were postponed trends, with an average delay of 0.06 d·a^−1^–0.24 d·a^−1^. The characteristics of EOS variation were mainly reflected in spatial differences (Fig. 4, Table 3). In terms of different aspects, the north EOS was the latest, the west was the earliest, the south and the north EOS always postponed, while the east and the west were mostly in advance.

However, from a statistical analysis, LST had no significant effect on EOS (Table 5), and changes in EOS might be related to the combined response of temperature and precipitation^9^, as studies of phenological phenomena in Europe and the United States have shown that autumn precipitation has a significant effect on EOS^31,32^ and reveals that precipitation affects EOS more strongly than temperature^33^. The LOS change showed a prolonged trend, increasing by an average of 0.79 d·a^−1^, which is consistent with the characteristics of phenological indicator changes in global warming signals study^34^. From the details of different aspects, the variation of LOS in the shady slpoe was larger than that of the sunny aspect, the average shortening of the south was 0.56 d·a^−1^, while the average shortening of the was 1.26 d·a^−1^. Especially, in 2001, the south LOS was as long as 166 d, while the west was only 135 d, the difference is 31d, this phenomenon also occurred in the study of RSP in the forest of Qilian Mountain because Long-term records of vegetation phenology suggest that temperature sensitivity can vary in space and time^9,22,35^. Under the aspect control, the overall change trends of the RSP were basically the same in zone I and zone II, and there was no statistical difference in RSP between two adjacent latitudes in the *L. gmelinii* forest area. ANOVA analysis showed that there was no significant difference between the same RSP on the same aspect in different zones, and that the same aspect would not have obvious effect on the RSP of *L. gmelinii* when the latitude span was 2°.

### Impact of LST on RSP

During the study period, there was little difference in the mean value of LST in different aspects, with a range of only 0.04 °C, but the CV was greater than 32%, indicating a great difference between years. The distribution characteristics of LST were that the sunlit aspect (east and south aspect) is slightly higher than the ubac aspect (west and north aspect). According to the effect of LST on SOS of *L. gmelinii*, LST contributed the most in April and spring, because they corresponded with SOS in time matching. Although the simultaneous (or early) precipitation also has an impact on SOS^22^, this effect is secondary to the impact of LST in the corresponding period of phenology^24^. None of the relationships of EOS to LST on different time scales have reached a statistically significant level. However, many phenological studies have shown that EOS is more affected by precipitation wherever it is^36^, such as the semi-arid prairie region of the Rocky Mountains that precipitation plays a more pronounced role than in humid temperate regions^37^, and same phenomenon also is found in the Tibetan Plateau of China as a major factor affecting EOS^38^. LOS was significantly affected by the mean spring and the April LST similar to the SOS vs. temperature factor. This means that the LOS prolonged is mainly affected by the advance of SOS, and the postponement of EOS does not contribute much to prolong LOS. This result can be confirmed in study on temperature and phenology in Northeast China^39^. In addition, the regression analysis of LST and RSP with significant at 0.001 level showed that the RSP’s of *L. gmelinii* on different aspects were very sensitive to the LST response at a specific temporal period (Fig. 6), This phenomenon can be verified in studies of different regions and different vegetation types^40–42^.

### Reliability analysis of RSP determined by M-K method

**Figure 8:**
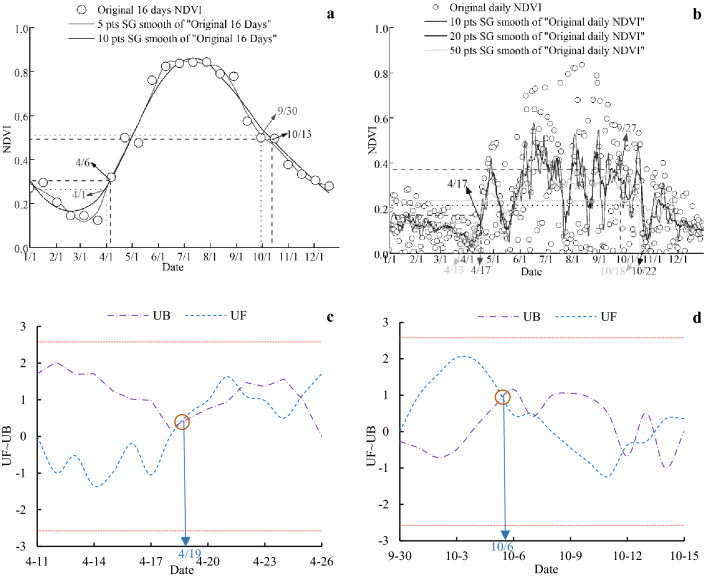
Comparison of RSP judgment between M-K and S-G smoothing methods (2019).

Because M-K method is rarely used in RSP determination, Savitzky Golay (S-G) method (SOS = NDVI_left min_ + (NDVI_max_-NDVI_left min_)*0.2; EOS = NDVI_right min_ + (NDVI_max_-NDVI_right min_)*0.4) is widely used in RSP^24^, so it is particularly important to compare the results of M-K and S-G in the determination of RSP of *L. gmelinii*. Thus, we chose different NDVI data of 2019 as a case analysis, which the average annual LST of 3.33 °C is almost similar to the average LST of 3.30 °C in study period. From the perspective of LST that have a great influence on RSP, this year is representative^21,22,36^. S-G analysis showed that the SOS corresponding to 5-point and 10-point smoothing was April 1 and 6, and the EOS was September 30 and October 13, respectively under the premise of 16-day NDVI data (Fig. 8a). Meanwhile, 5-point smoothing has almost no effect and presents a similar discrete state to the original NDVI under the premise of using daily NDVI data for identifying SOS (Fig. 8b); and 10, 20 and 30 points smoothing were April 17, April 17 and April 13 for identifying SOS respectively, and the EOS were September 27, October 22 and October 18 for identifying SOS respectively. The M–K test method used to determine the RSP result is that SOS appeared on April 9 and EOS appeared on October 6 (Fig. 8c,d). The M–K test method used to determine the RSP result is that SOS appeared on April 9 and EOS appeared on October 6 (Fig. 8c,d).

From the mutual verification of the above results and the existing findings, Yang et al. (2016) studied the phenology of *L. gmelinii*. in the Greater Khingan Mountains from 1987 to 2012 and showed that the flower bud opening time (SOS) appeared as early as April 17 (1992) and as late as May 15 (1987). The earliest date of full leaf discoloration (EOS) is September 18 (1990), and the latest date is November 8 (2012)^43^. Therefore, according to the comparison of SOS results (Table 6), the S-G method by using the 16-day NDVI data to determine RSP of *L. gmelinii* is earlier with lower credibility than the NDVI daily data in the Greater Khingan Mountains. From the comparison of EOS results (Table 6), the S-G methods by using different data sources are all within the variation range of Yang's observation results. Finally, results of M–K test method of this paper, both SOS and EOS are within the observation range of Yang. Therefore, it could be confirmed that using NDVI to determine the RSP of *L. gmelinii*, whether using S-G or M–K test method, the daily NDVI data is better than the 16-day. In addition, S-G method also has the influence of different number of smoothing points on the results, whereas M–K test method does not have this uncertainty, which enhances the comparability of different research findings.

**Table 6:** Comparison of results of different data sources and different RSP determination methods (Month/day).

RSP	16d NDVI S-G		Daily NDVI S-G			Daily NDVI
	5 pts smooth	10pts smooth	10 pts smooth	20 pts smooth	50 pts smooth	M–K test
SOS	4/1	4/6	4/17	4/17	4/13	4/19
EOS	9/30	10/13	9/27	10/22	10/18	10/6

However, large-scale vegetation RSP is affected by vegetation type and species composition. In this study, the *L. gmelinii* forest area is associated with evergreen coniferous trees such as *P. koraiensis* and *P. sylvestris* var. *mongolica*, as well as summer green broad-leaved trees such as *B. platyphylla*, and a certain area of alpine meadows is also distributed, then, the uncertainty of NDVI variation caused by species composition and ecosystem differences results insufficient in the accuracy of the research findings. This phenomenon has been clearly pointed out in the study of the phenological pattern of The Greater Khingan Mountains^44,45^, and the difference in phenological determination methods could also lead to uncertainty in RSP. So it is necessary to strengthen the comparison of research methods and corresponding interpretation.

## Conclusion

The RSP of *L. gmelinii* is affected by the aspect. The SOS of the east (0.71d a^−1^) and the west (1.02d a^−1^) advanced more than that of the southern aspect (0.50 d a^−1^) and the Northern Aspect (0.46d a^−1^). EOS is postponed except for the east, but the magnitude is small. LOS is prolonged on all aspects, the longest on the west (1.26d a^−1^) and the shortest on the south (0.56 d a^−1^). SOS occurs in the order of the west > the north > the south > the east. The EOS order is the north > the south > the east > the west. LOS follows the order of the south > the north > the east > the west.

LST has a significant direct effect on the phenological changes of *L. gmelinii* in different aspects, the influence of LST on SOS and LOS is obviously greater than that on EOS. LST has significant effects on SOS and LOS in April and spring (*P* < 0.001), that is, the higher the LST is, the earlier SOS occur and the longer duration of LOS. The main contributor to the increase of LOS is the advance of SOS, while the prolonged of EOS has a relatively small contribution to LOS.

## Supplementary Information

Supplementary Information 1.

Supplementary Information 2.

Supplementary Information 3.

Supplementary Information 4.

Supplementary Information 5.

Supplementary Information 6.

Supplementary Information 7.

Supplementary Information 8.

Supplementary Information 9.

Supplementary Information 10.

Supplementary Information 11.

## Data Availability

All data and supporting data associated with the conception and experiments is available in the supporting information.
